# The Effect of Traditional and Cyclodextrin-Assisted Extraction Methods on *Trifolium pratense* L. (Red Clover) Extracts Antioxidant Potential

**DOI:** 10.3390/antiox11020435

**Published:** 2022-02-21

**Authors:** Jurga Andreja Kazlauskaite, Liudas Ivanauskas, Mindaugas Marksa, Jurga Bernatoniene

**Affiliations:** 1 Department of Drug Technology and Social Pharmacy, Lithuanian University of Health Sciences, LT-50161 Kaunas, Lithuania; jurga.andreja.kazlauskaite@lsmu.lt; 2 Institute of Pharmaceutical Technologies, Lithuanian University of Health Sciences, LT-50161 Kaunas, Lithuania; 3 Department of Analytical and Toxicological Chemistry, Lithuanian University of Health Sciences, LT-50161 Kaunas, Lithuania; liudas.ivanauskas@lsmuni.lt (L.I.); mindaugas.marksa@lsmu.lt (M.M.)

**Keywords:** red clover, cyclodextrins, antioxidants, polyphenols, oxidative stress

## Abstract

Red clover is the subject of numerous studies because of its antioxidant properties, the positive influence of isoflavones on the health, and its potential use in the prevention and treatment of chronic diseases. The right excipients, such as cyclodextrins, can increase the profile of valuable phenolic compounds in extraction media to obtain rich in antioxidants, extracts that can be used in nutraceuticals production. The aim of this study was to investigate and compare the total phenolic content, flavonoid content, and antioxidant activity of red clover aerial parts, aqueous and ethanolic extracts prepared using traditional and cyclodextrins-assisted methods. The antioxidant activity of the extracts was established using ABTS, DPPH, FRAP, and ABTS-post column methods. It was determined that cyclodextrins significantly increased total phenolic content (compared with control)—using *β*-cyclodextrin 20.29% (in aqueous samples); *γ*-cyclodextrin 22.26% (in ethanolic samples). All the samples prepared with excipients demonstrated a strong relationship between total phenolic content and DPPH assay. Study showed that for extraction with water, the highest amounts of phenolic compounds, flavonoids and antioxidant activity will be achieved with *β*-cyclodextrin, but extractions with ethanol will give the best results with *γ*-cyclodextrin. Therefore, cyclodextrins are a great and safe tool for obtaining rich, red clover flower extracts that are high in antioxidant activity, which can be used in the pharmaceutical industry for nutraceuticals production.

## 1. Introduction

Red clover (*Trifolium pratense* L.) has high concentrations of phenolic compounds, especially isoflavonoids, which are largely distributed in the *Leguminosae* family [1]. Its extracts are rich in phenolic compounds such as flavonoids, phenolic acids, clovamides, and saponins [2]. Scientific research determined, that red clover extracts consists of 35.54% isoflavones, 1.11% other flavonoids, 0.06% pterocarpans, ≤0.03% coumarins, and ≤0.03% tyramine [3]. Flavonoids are an important class of phenolic compounds that includes flavones, flavonols, and isoflavones, all characterized by a phenylbenzopyran moiety [4]. Isoflavones are a group of flavonoids typical of some legume species only. They exhibit estrogenic activity and represent the main phytoestrogens of current interest as nutraceuticals and dietary supplements. An antioxidant activity of possible physiological relevance was also reported for isoflavones such as genistein and daidzein [5,6,7,8]. The role of flavonoids (isoflavones) and other phenolic compounds as protective dietary constituents, with their estrogenic, antimicrobial, anti-inflammatory, antiallergenic, and antitumor activities, have become an increasingly important area of research. These compounds are useful in nutraceuticals, cosmetics, medicine, and food additives, or used in the agri-food industry [9,10,11].

Phenolic compounds, including flavonoids, are antioxidants due to their ability to scavenge and/or reduce high-energy radicals. It is confirmed that total phenolic content correlates with antioxidant activity [12,13]. The antioxidant activity of phenolic compounds is mainly due to their redox properties, which can play an important role in neutralizing free radicals. Antioxidant properties stand to be a valuable mechanism of beneficial activity of plant-derived compounds and extracts [14,15,16,17,18].

There are numerous articles confirming antioxidant activity of *Trifolium pratense* L. The results of research suggested the great value of flowers and leaves of *Trifolium* species for their use in phytotherapy, due to their content of polyphenols responsible for the antioxidant activities; phenols in plants can also act as deterrents against herbivores, as antiviral and antimicrobial factors and photoprotectants against UV irradiation [19,20,21]. Using the right extraction method more phenolic compounds can be extracted, therefore, the antioxidant activity can be increased. Additional materials, such as excipients, may be used to extract insoluble polyphenols such as daidzein and genistein as well as enrich the extract with biologically active compounds [22]. 

The initial selection of herbal compounds possessing the antioxidant effects is carried out by in vitro methods. Spectrophotometric methods are widely applied [23,24,25,26]. They determine total amount of antioxidants and evaluate the total antioxidant activity in the researched complex samples. Most popular methods are scavenging 2,2-diphenyl-1-picrylhydrazyl (DPPH•) and/or 2,2’-azino-bis(3-ethylbenzothiazoline-6-sulfonic acid) (ABTS•+) free radicals [27]. The most reliable and comprehensive evaluation of the radical-scavenging capacities of plant materials is possible only through the validation of highly efficient analytical methods. One highly promising method for the investigation of antioxidant activity is the HPLC post-column assay with various derivatization techniques. These methods exclude the interactional effects of compounds as the detection occurs with separated analytes. Most post-column assays use DPPH and ABTS radicals [28,29].

Excipients play important role in dosage form. These compounds are added to the formulation along with pharmacologically active substance. Almost all drug dosage forms include excipient to guarantee the dosage, stability, and bioavailability [30]. Cyclodextrins (CDs) can be suitable as excipients in excipient-assisted extraction [31]. It is generally accepted that CDs can form inclusion complex in aqueous solution where a lipophilic guest molecule or moiety locates in the inner cavity [32]. Due to this special property, CDs have been used extensively in pharmaceutical research; CDs can address solubility, stability, and bioavailability issues in a manner not possible with other inactive ingredients. CDs can also, for example, be used to convert liquid drugs into microcrystalline powders, prevent drug-drug or drug additive interactions, reduce or eliminate unpleasant taste and smell, and improve shelf-lives. The most common cyclodextrins are *α*-CD, *β*-CD, and *γ*-CD, which consist of six, seven, and eight glucopyranose units, respectively [33,34,35]. 

To our knowledge, this is the first article describing the effect of CDs on the antioxidant activity of red clover flowers extracts. Therefore, the aim of the study was to investigate and compare total phenolic content and antioxidant activity of red clover aerial parts extracts prepared using traditional methods and using CDs as excipients. We also seek to examine the effects of excipients on total flavonoids content. 

## 2. Materials and Methods

### 2.1. Plant Material and Reagents

Red clover samples were collected in red clover fields in Laičiai, Kupiškis district, Lithuania (latitude 55°53024.2″ N; longitude 25°19036.0″ E). The collections of flower buds and flowers were made on the 26 September 2020. Samples were dried and stored at room temperature. Before use, clover flowers were ground to a fine powder using an Ultra Centrifugal Mill ZM 200 (Retsch, Haan, Germany). Grinding was performed at 4025 g using a 0.5 mm trapezoid hole sieve. 

Purified water was prepared with GFL2004 (GFL, Burgwedelis, Germany). Deionized water was prepared with Milipore, SimPak 1 (Merck, Darmstadt, Germany). The following reagents were used: 2,2′-azino-bis(3-ethylbenzothiazoline-6-sulfonic acid) (ABTS), 2,4,6-Tris(2-pyridyl)-s-triazine (TPTZ), aluminium chloride, hexaethylenetetraamine, acetic acid obtained from Sigma-Aldrich (Buchs, Switzerland); potassium persulfate from Alfa Aesar (Karlsruhe, Germany); monosodium phosphate, ferrous sulfate heptahydrate, saline phosphate buffer and hydrogen peroxide from Sigma Aldrich (Schnelldorf, Germany); disodium hydrogen phosphate obtained from Merck (Darmstadt, Germany); 2,2-diphenyl-1-picrylhydrazyl radical (DPPH) and *α*-, *β*-, and *γ*-CDs purchased from Sigma Aldrich (Hamburg, Germany); ethanol (96%) (Vilniaus Degtinė, Vilniaus, Lithuania).

### 2.2. Extracts Preparation

The raw material of red clover aerial parts was ground, and the moisture content of the milled red clover flowers was determined using a KERN MLB apparatus (KERN & Sohn GmbH, Balingen, Germany). The production of extracts was performed as described by Kazlauskaite et al. [22]. 

Heat-Reflux Extraction (HRE) was performed by using 0.3 ± 0.001 g of dried and milled flower heads. Plant material was mixed with 10 mL of solvent (50% ethanol v/v or purified water) in a round bottom flask and refluxed in a sand bath at 100 °C for 1 h. After reflux, the sample was left to cool down at a temperature of 25 ± 2 °C. After cooling down, the sample was centrifuged with Sigma 3-18K centrifuge (Sigma, Osterode am Harz, Germany) for 10 min at 3382× *g*, followed by the decantation of the supernatant. The extract was filtered through PVDF syringe filters (pore size 0.22 µm). 

Ultrasound-assisted extraction with thermal hydrolysis (UAE) was performed using an ultrasound bath (frequency 38 kHz) (Grant Instruments™ XUB12 Digital, Cambridge, England). The extraction was performed by using 0.3 ± 0.001 g dried and milled flower heads. Plant material was macerated in 10 mL of solvent, and the extraction of isoflavones using ultrasound was performed by employing different conditions: solvent (50% *v*/*v* ethanol and purified water) and extraction time (10 or 30 min), with the processing temperature of 40 ± 2 °C (the temperature is regulated automatically by the ultrasonic bath). After ultrasound processing the mixture was prepared for thermal hydrolysis. The sample was transferred to a 250 mL round bottom flask. It was refluxed in a sand bath at 100 °C for 1 h. After the procedure, the mixture was left to cool down and then centrifuged for 10 min at 3382× *g*, followed by the decantation of the supernatant. The extracts were filtered through PVDF syringe filters (pore size 0.22 µm, Frisenette, Knebel, Denmark).

Some of the samples were prepared with excipients: *α*-, *β*-, or *γ*-CDs. The extracts were made under the same conditions as previously listed (HRE or UAE). Purified water or 50% ethanol (*v*/*v*) was used as the solvent (10 mL), and the excipient (0.1 ± 0.001 g) was added to the extraction mixture with plant material (0.3 ± 0.001 g). CD concentrations in the sample was 1% (*w*/*v*). The excipient amount was based on the quantity of solvent in the extract. The samples were centrifuged for 10 min at 3382 × *g*, followed by the decantation of the supernatant. After that, the extracts were filtered through PVDF syringe filters (pore size 0.22 µm). 

**Figure 1:**
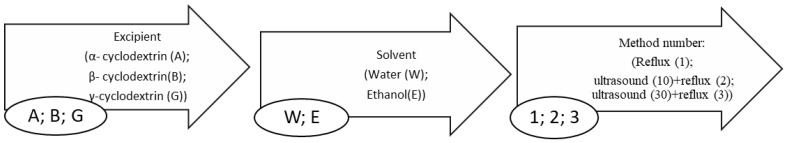
Sample codes generation scheme. If the excipient was not used in the preparation of the sample, the letter of excipient will be skipped, and the code will start from the solvent letter.

A list of samples prepared using different conditions and excipients is given in Table 1. For easier comprehension samples codes are based on their preparation conditions and decoding is shown in Figure 1.

### 2.3. Determination of Total Phenolic Content

The content of total phenolic compounds in red clover samples was determined by using a slightly modified Saviranta et al. method [36], so 0.5 mL of extract was mixed with 2.5 mL of 1:9 diluted Folin–Ciocalteu’s phenol reagent and 2.0 mL of 7% (*w*/*v*) sodium carbonate. Absorbance was measured at 765 nm after 1 h using a spectrophotometer (Shimadzu UV-1800, Kyoto, Japan). The calibration curve was obtained with a gallic acid (0–0.1 mg/g; y = 11.108; R^2^ = 0.9981). The results were expressed as gallic acid equivalent per gram dry weight (mg GA/g dw).

### 2.4. Determination of Total Flavonoid Content

Analysis of total amount of flavonoids in extracts was performed by UV spectrophotometry, based on complexation of phenolic compounds with Al(III), as described in Urbonavičiute et al. with some modifications [37]. Accordingly, 0.1 mL of red clover aerial part extract was added to 1.0 mL 96% (*v*/*v*) of ethanol, 0.05 mL of 33% acetic acid, 0.15 mL 10% aluminium chloride, and 2.0 mL 5% hexaethylenetetraamine solutions. Spectrophotometric analysis was performed after 30 min at 475 nm wavelength using aspectrophotometer (Shimadzu UV-1800, Kyoto, Japan). The results were expressed as rutin equivalent per gram dry weight (RE/g dw) and calculated by the following formula: TFC = C × V_e_ × F/M, where TFC is the total flavonoid content, mg RE/g dw; C is the concentration of the used standard, mg/L; V_e_ is the volume of the used solvent, L; F is the dilution coefficient of the sample; and M is the mass of the sample, g. The calibration curve was obtained with a rutin (0–0.5 mg/g; y = 5.0867; R^2^ = 0.9985). The results were expressed as rutin equivalent per gram dry weight (RU/g dw).

### 2.5. Antioxidant Activity

#### 2.5.1. Ferric Reducing Antioxidant Power (FRAP)

The FRAP assay was prepared by mixing 0.3 M acetate buffer, 10 mM TPTZ solution with 40 mM HCl and 20 mM ferric chloride solution. Then, 10 μL of sample was mixed with 200 μL of FRAP reagent; the contents were mixed vigorously. The absorption was measured at 593 nm using spectrophotometer (Shimadzu UV-1800, Kyoto, Japan). The calibration curve was obtained with a ferrous sulfate (0–1 mg/g; y = 2.6272; R^2^ = 0.9985). The results were expressed as ferrous sulfate equivalent per gram dry weight (FE(II)/g dw)

#### 2.5.2. ABTS Radical Scavenging Activity Assay

The ABTS radical was generated through the oxidation of ABTS with potassium persulfate. Aqueous ABTS solution (7 mM) was mixed with potassium persulfate (2.45 mM) solution and stored in the dark for 12–16 h to produce a dark-colored solution containing ABTS radical cation. Before use, the ABTS radical cation was diluted with water with an initial absorbance of about 0.90 (±0.02) at 734 nm using spectrophotometer (Shimadzu UV-1800, Kyoto, Japan). Free radical scavenging activity was evaluated by mixing 2.0 mL of ABTS working standard with 200 μL of test sample in cuvette. The samples ware incubated in the dark at room temperature for 30 min. The calibration curve was obtained with a trolox (0–0.5 mg/g; y = 0.0001728x; R^2^ = 0.9832). The results were expressed as trolox equivalent per gram dry weight (TE/g dw) [38].

#### 2.5.3. DPPH Radical Scavenging Activity Assay

Here, 2.0 mL of DPPH solution (0.1 mM, in ethanol) was blended with 2.0 mL of the samples. The reaction mixture was shaken and incubated in the dark at room temperature for 30 min, and the absorbance was read at 517 nm against the blank using spectrophotometer (Shimadzu UV-1800, Kyoto, Japan). The calibration curve was obtained with a trolox (0–0.016 mg/g; y = 0.00623x; R^2^ = 0.9923). The results were expressed as trolox equivalent per gram dry weight (TE/g dw) [39]. 

### 2.6. ABTS Post-Column Antioxidant Assay

The HPLC post-column method using ABTS reagent was carried out according to Marksa et al. [29].

**Figure 2:**
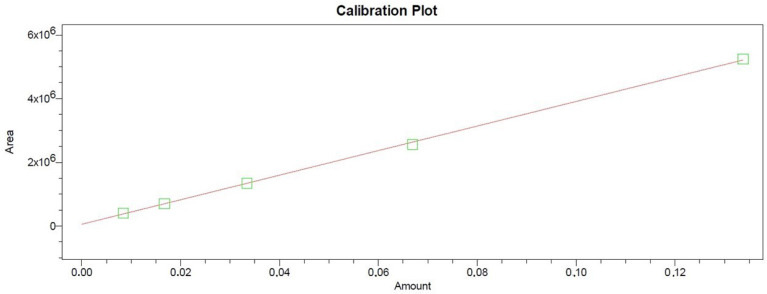
Trolox calibration curve.

The samples were introduced into the HPLC detection system, the mobile phase with test sample passes through the reaction loop into the mixing tee, where a 0.5 mL/min ABTS reagent solution is supplied simultaneously via a Gilson pump 305 (Middleton, WI, USA). The HPLC-ABTS system uses a 3 m (inner diameter 0.25 mm, outer diameter 1.58 mm) reaction loop, which was thermostated at 50 °C. Reaction of antioxidants with ABTS resulted in a color change of the reagent as determined using a Waters 2487 UV / VIS detector (Waters Corporation). Detection of test solutions was performed at a wavelength of 650 nm. Signal strength is expressed as peaks of negative active compounds. The antioxidant activity of the extract compounds was evaluated by the post-column method according to the equivalent of the trolox standard. Calibration curves were prepared using trolox ethanol solution. An ACE 5 C18 250 × 4.6 mm column (Advanced Chromatography Technologies, Aberdeen, Scotland) was used. The mobile phase consisted of solvent A (acetic acid/methanol/deionized water) (1:10:89 *v*/*v*/*v*) and solvent B (acetic acid/methanol) (1:99 *v*/*v*/*v*). The linear gradient elution profile was as follows: 80% A/20% B at 0 min; 30% A/70% B at 30 min; 90% A/10% B at 39 to 40 min. The flow rate was 1 mL/min, and the injection volume was 10 µL. Calibration curve was prepared from a trolox ethanol solution at five dilutions in the range of 8.359–133.750 μg/mL, R^2^ = 0.999565 (Figure 2). 

### 2.7. LC-MS Qualitative Analysis

After performing reversed-phase liquid chromatographic (RP-LC) separation, the detailed qualitative profiling of red clover extract was carried out using electrospray (ESI) ionization (in negative and positive mode) followed by the mass spectrometric (MS) analysis. The LC/MS system was composed of a Shimadzu Nexera X2 LC-30AD HPLC system (Shimadzu, Tokyo, Japan) equipped with a LCMS-2020 mass spectrometer (Shimadzu, Tokyo, Japan).

The chromatographic separation was performed on a YMC-Triart C18 (YMC Karasuma-Gojo, Kyoto, Japan) (150 mm × 3.0 mm, 3 μm) analytical column, column temperature 40 °C. The mobile phase A consisted of 0.1% formic acid in water, and mobile phase B consisted of 0.1% formic acid in acetonitrile. For each clover sample, 10 μL aliquots volumes were injected onto the chromatographic column. HPLC was run at 0.4 mL/min flow. The optimum ESI conditions were set as 350 °C for interface temperature, 250 °C for DL temperature, 400 °C for heat block temperature, 1.5 L/min for nebulizing gas flow, and 10 L/min for drying gas flow. Positive ion and negative ion measurements are performed while switching alternately between positive and negative ionization modes. The *m*/*z* ranges for positive and negative modes were 50–1000, scan speed 15,000 u/s, and 0.1 *m*/*z* steps. 

Compounds present in the sample were identified by comparing the mass spectra obtained with both the literature data and mechanisms presented in freely available databases.

### 2.8. Statistical Data Analysis

Data were analysed using SSPS version 20.0 (IBM Corporation, Armonk, NY, USA). All experiments were performed in triplicate. Data are expressed as mean ± standard deviation (S.D.). The comparisons between three different measurements were made using Friedman and Wilcoxon tests. Comparisons between the two groups were made by the Mann–Whitney U test. Correlation and regression coefficients were performed using the Spearman test. The results were considered statistically significant at *p* < 0.05.

## 3. Results and Discussion

### 3.1. The Influence of Excipients on the Total Amount of Phenolic Compounds and Flavonoids Content

There is very little information on the total phenolic and flavonoid compounds in the flower extracts of *Trifolium pratense* L. Extracts of red clover leaves have been studied the most, as they were thought to contain the highest amounts of isoflavones, which makes clover very valuable [40,41,42]. However, further research has shown that clover flowers also contain significant amounts of isoflavones [43,44,45]. In the previous research, the optimal extraction conditions were determined to conceive high isoflavones daidzein and genistein yields from red clover aerial parts [22]. In this study, these conditions were used to study the effects of CDs on total phenolic compounds, flavonoids content, and antioxidant activity in *Trifolium pratense* L. flowers extracts.

Antioxidants, including phenolic compounds (e.g., flavonoids, phenolic acids), have diverse biological effects. The antioxidant extracts were evaluated in terms of their total phenols and total flavonoids. Being plant secondary metabolites, polyphenols are very important judging from the virtue of their antioxidant activities by chelating redox-active metal ions, inactivating lipid free radical chains and avoiding the hydroperoxide conversions into reactive oxyradicals [2]. 

**Figure 3:**
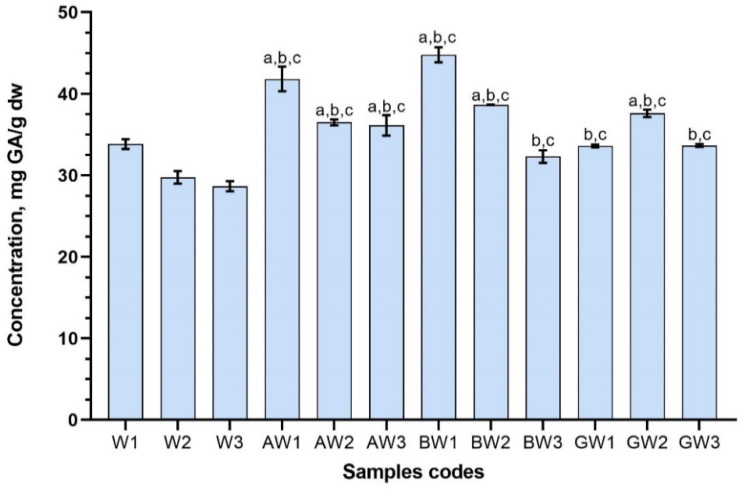
Total phenolic content in red clover flowers extracts with excipients using water as a solvent. Results are mean values (n = 3). ^a^ *p* < 0.05 vs. W1; ^b^ *p* < 0.05 vs. W2; ^c^ *p* < 0.05 vs. W3. Samples codes are provided in Table 1.

**Figure 4:**
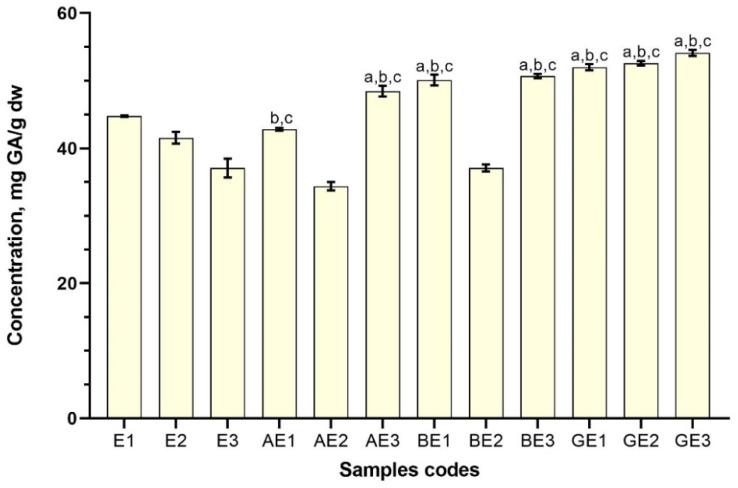
Total phenolic content in red clover extracts with 50% ethanol (*v*/*v*) as a solvent (n = 3). Results are mean values (n = 3). ^a^ *p* < 0.05 vs. E1; ^b^ *p* < 0.05 vs. E2; ^c^ *p* < 0.05 vs. E3. Samples codes are provided in Table 1.

The total phenolic contents in samples of the extracts, expressed as gallic acid equivalents, varied from 44.78 ± 0.92 (BW1) to 32.31 ± 0.63 (BW3) mg GA/g dw when the solvent was water (Figure 3), and 54.12 ± 0.46 (GE3) to 34.38 ± 0.63 (AE2) mg GA/g dw when the solvent was 50% (*v*/*v*) ethanol (Figure 4). Most of the samples that were prepared with excipients, despite the solvent used, had illustrated higher total content of phenolic compounds compared with control samples prepared without CDs (*p* < 0.05).

Using water as a solvent the highest phenolic compound content was in sample BW1 (44.78 ± 0.92 mg GA/g dw), very similar amount was detected in sample AW1 (41.82 ± 1.51), that was prepared using same conditions as BW1 (Figure 3). The results showed that when using excipients in aqueous extractions, phenolic compound content increased significantly (*p* < 0.05) compared to that of control samples. Using *α*- or *β*-CDs, the most effective extraction method for phenolic compound extraction was reflux but combining it with ultrasound decreased phenolic compounds yield. Using *γ*-CD as an excipient and combining ultrasound processing with reflux was most effective, but the yield of total phenolic content obtained was lower than using *α*- or *β*-CDs (Figure 3). There were three samples (BW3; GW1; GW3) that were not significantly higher than control sample W1. This may have been since reflux alone was not sufficient with *γ*-CD, but the GW2 sample after sonication showed good results. Therefore, using sonication increases the phenolic compounds content, but by prolonging it from 10 to 30 min the amount of compounds decreases. The hypothesis can be made that using ultrasound for a longer period causes degradation of the phenolic compounds in the extract. This case was observed not only with *γ*- but also with *β*-CDs samples in water (BW3; GW3).

A lot of information can be found on the effect of solvent on the extraction of antioxidant phenolic compounds from different raw materials. The results of control samples (W1-3 and E1-3), that were prepared using the same conditions, but different solvents (water and 50% ethanol (*v*/*v*)) show that more phenolic compounds are recovered from red clover blossoms using ethanol as solvent. However, using water and CDs as excipients in the extraction increases the total amount of phenolic compounds. The use of CDs in an aqueous solution as extraction media is considered to be a green extraction since water is the main solvent and the existence of CD hydrophobic cavity boosts the extraction of phenolic compounds due to the formation of the inclusion complex [22].

Using 50% ethanol (*v*/*v*) as the solvent with excipients, it was determined that the highest content of phenolic compounds was found in the sample GE3 (54.11 ± 0.45 mg GA/g dw) (Figure 4). *γ*-CD was the most effective excipient used in ethanol compared to the results obtained from samples with *α*- and *β*-CDs. The best results for *β*-CD in ethanol were obtained using the same conditions as with water; the total amount of phenolic compounds was 50.11 ± 0,79 mg GA/g dw (BE1). Using ethanol with α-CD the best results was acquired employing ultrasound processing for 30 min and thermal hydrolysis—48.46 ± 0.80 mg GA/g dw (AE3). 

All the results, except AE1, AE2, and BE2, were statistically significantly higher (*p* < 0.05) compared to the control samples. In ethanol, using *α*-CD and reflux or reflux combined with ultrasound processing for 10 min results decreased and had the opposite effect compared to *α*-CD samples prepared in water. However, increasing ultrasound-processing time to 30 min the total phenolic content increased. A similar case has been observed with *β*-CD as well. *γ*-CD in ethanol had the best extraction properties compared to other CDs.

Comparing total phenolic content results obtained from samples with different solvents (water and 50% ethanol (*v*/*v*)), it was observed that some of the aqueous test samples, prepared using an excipients-assisted method, showed higher total phenolic compounds content than ethanol samples with the same excipients. Aqueous samples obtained using *α*- or *β*-CDs excipients, sonicated for 30 min and then hydrolysed in high temperature, extracted more phenolic compounds than 50% ethanol. In addition, 6.17% more phenolic compounds were found in sample AW2 than in AE2 and in BW2—4.21% more than in BE2. The largest difference that was observed in this study was between samples GE3 and GW3—37.80%, but in this case ethanolic sample GE3 was higher than aqueous GW3.

The content of total flavonoids ranged from 21.74 ± 0.12 to 17.95 ± 0.08 mg RU/g dw using excipients and water as a solvent. Using excipients and ethanol as a solvent, the total flavonoids content ranged from 21.18 to 20.82 mg RU/g dw (Table 2). The total flavonoid content results were entirely synchronous with those of the total phenolic content. It was successfully shown that samples with a high level of phenolic content also contain flavonoids in a great amount. Plants rich in flavonoids can be a good source of antioxidants to help increase the body’s overall antioxidant capacity and prevent it from lipid peroxidation [46].

Samples AW1-3 and BW1-3 were significantly higher (*p* < 0.05) compared to all control samples W1-3. GW1-2 was statistically significantly higher only compared to control sample W1. Highest total flavonoid content was found in aqueous samples BW1 and BW3, which were prepared using *β*-CD (21.74 ± 0.12 and 21.47 ± 0.23 mg RU/g dw, respectively). The lowest flavonoids yields were detected in samples prepared with *γ*-CD. Using ethanol as a solvent, the highest flavonoid content was found in AE2 (21.18 ± 0.24 mg RU/g dw). Similar quantities of flavonoids were observed in AE1 (21.13 ± 0.13 mg RU/g dw), BE1 (21.11 ± 0.28 mg RU/g dw), and GE3 (21.09 ± 0.19 mg RU/g dw) samples. 

Biljana Kaurinovic et al. determined flavonoids content in red clover using water or ethanol was much lower comparing with the results obtained in this study [24]. However, as in our study, it was found that the use of water gives a similar yield of flavonoids as the use of ethanol.

Comparing samples in water and ethanol using CDs as excipients, reverse correlation was discovered between *γ*- and *β*-CDs depending on the solvent used. 

**Figure 5:**
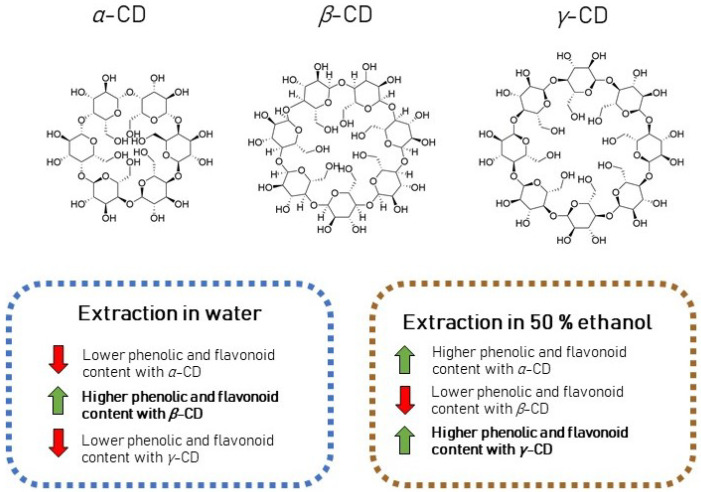
Comparison of the effect of excipients on the concentration of phenolic compounds using different solvents (water or 50% ethanol).

Using *γ*-CD in water yielded lower amounts of flavonoids than under the same conditions in ethanol. The use of *β*-CD in water gave significantly better results than its use in ethanol. Thus, *β*-CD helps to extract flavonoids in water. Solvents impact to *α*-CD was hardly noticed, but more flavonoids were extracted in ethanol with this excipient. The different effects of CDs in complex formation in different solvents are shown in Figure 5. 

### 3.2. Antioxidant Activity

The antioxidant potential of red clover flowers extract with CDs as excipients in extraction media was investigated by using different antioxidant activity methods: the ABTS and DPPH methods as well as the ferric reducing antioxidant power (FRAP) test. It is strongly recommended to use a broad spectrum of methods, given that plant extracts contain many different classes and types of antioxidants. The phytochemical composition of extracts is affected by the extraction conditions, and, in our case excipients (CDs), which strongly influence the antioxidant effects [47,48,49].

#### 3.2.1. DPPH Radical Scavenging Activity

In several studies, it has been reported that inclusion complex formation increases antioxidant capacity [49,50]. The presence of a guest molecule in a CD molecule results in increased electron density, resulting in a greater likelihood that the guest molecule will release protons as radicals to quench the DPPH radical [50].

**Figure 6:**
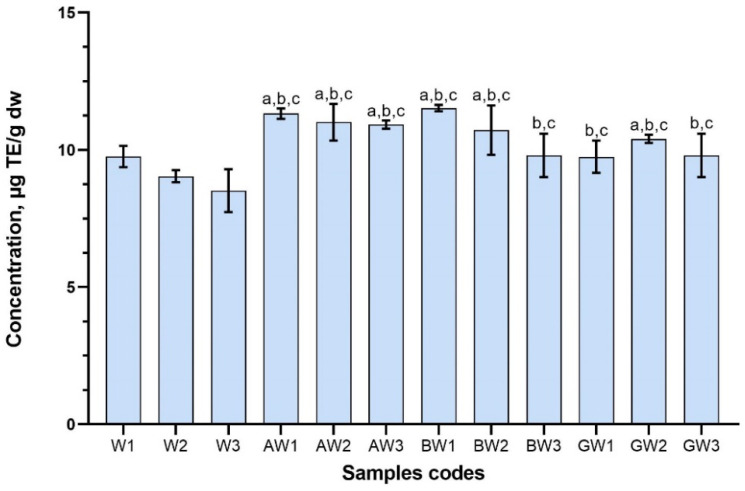
DPPH radical scavenging activity of aqueous red clover blossom extracts with excipients. Results are mean values (n = 3). ^a^ *p* < 0.05 vs. W1; ^b^ *p* < 0.05 vs. W2; ^c^ *p* < 0.05 vs. W3. Samples codes are provided in Table 1.

A DPPH scavenging activity was expressed as µg/g as trolox equivalent. DPPH discoloration, and a higher value signifies a higher antioxidant potential. In this research, significant differences (*p* < 0.05) between the antioxidant activities among the aqueous red clover samples with excipients and control samples were found (Figure 6). The DPPH radical scavenging in the tested aqueous samples varied from 9.75 ± 0.59 (GW1) to 11.52 ± 0.12 (BW1) µg TE/g dw. 

Only three samples (BW3, GW1 and GW3) did not have statistically higher antioxidant activity than control W1. The samples that contained highest total phenolic content had the strongest antioxidant activity using DPPH method. Therefore, CDs increase the quantity and/or concentration of phenolic compounds, which have strong antioxidant properties. 

**Figure 7:**
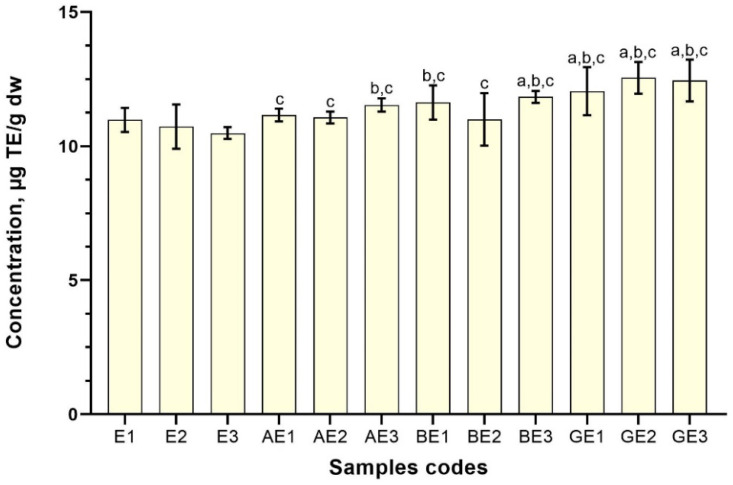
DPPH radical scavenging activity of red clover blossom extracts with 50% (*v*/*v*) of ethanol as a solvent with excipients. Results are mean values (n=3). ^a^ *p* < 0.05 vs. E1; ^b^ *p* < 0.05 vs. E2; ^c^ *p* < 0.05 vs. E3.

By using the DPPH assay on extracts prepared with 50% ethanol (*v*/*v*), CDs demonstrated the greatest radical scavenging activity—12.55 ± 0.59 µg TE/g dw in the sample GE2 and 12.45 ± 0.78 µg TE/g dw in the sample GE3 (Figure 7). These results are in good agreement with the phenolic and flavonoid contents in Figure 4 and Table 2. Using *α*-CD in the ethanolic red clover flowers extract antioxidant activity was weaker compared to extracts where *β*- or *γ*-CDs were used. Samples AE1 and AE2 were only significantly higher (*p* < 0.05) compared to the E3 control sample. Prolonging ultrasound from 10–30 min increased antioxidant activity in the AE3 sample that was statistically higher compered to E2 and E3 control samples. 

Previous studies that used concentrated ethanol as a solvent reported that red clover extract in DPPH assay gave a value at 3.7 μg TE/g [51]. The results obtained in this study were significantly higher even in controls without excipients, compared to reported results in other articles, indicating that water or ethanol are suitable solvents for the extraction of compounds with strong antioxidant effects from red clover aerial parts.

#### 3.2.2. ABTS Radical Scavenging Activity

The ABTS radical cation decolorization test is one of the most widely used methods to evaluate antioxidant activity. Color reduction shows the decrease in the ABTS radical [2]. 

**Figure 8:**
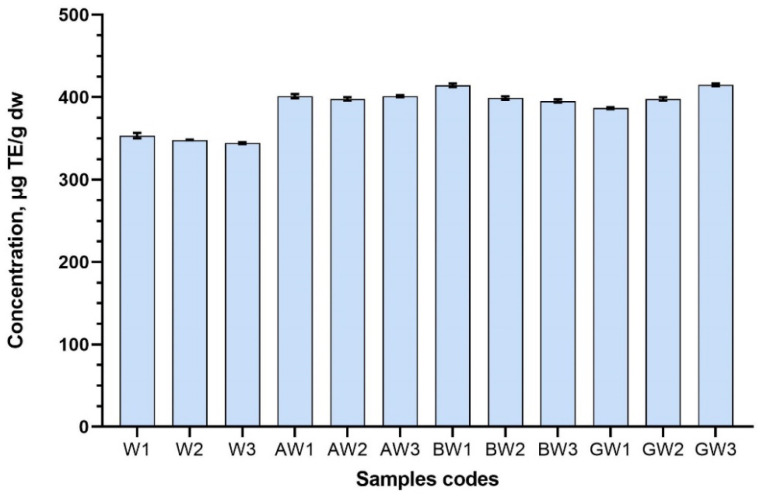
ABTS radical scavenging activity of aqueous red clover blossom extracts with excipients. Results are mean values (n = 3). All the test samples were statistically significant to controls, therefore, comparisons are not marked with letters in the graph. Samples codes are provided in Table 1.

In this study, all the test samples prepared with CDs were significantly higher compared with control samples. The highest antioxidant activity (in samples where the solvent was water) after the reaction with ABTS assay was observed in sample BW1 (414.53 ± 2.09 μg TE/g) and GW3 (415.01 ± 1.45 μg TE/g) (Figure 8). From all the test samples (excluding control samples), the lowest antioxidant activity was observed in sample GW1 (386.768 ± 1.25 μg TE/g) (Figure 8). GW1 was the only sample that was statistically significantly smaller compared with all the test samples prepared with excipients. The ABTS radical scavenging capability using excipients of the test samples can be ranked, according to their average means, as follows: BW > AW > GW. 

**Figure 9:**
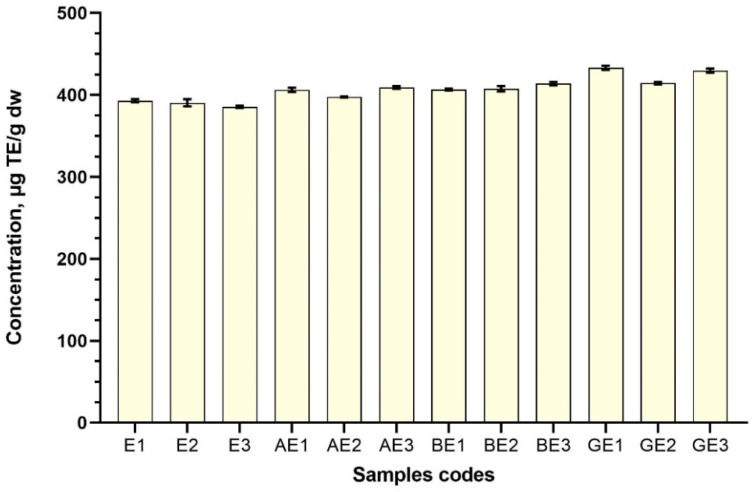
ABTS radical scavenging activity of ethanolic red clover blossom extracts with excipients. Results are mean values (n = 3). All the test samples were statistically significant to controls, therefore, comparisons are not marked with letters in the graph. Samples codes are provided in Table 1.

The highest antioxidant activity (in samples where the solvent was 50% ethanol (*v*/*v*)) was detected in the sample GE1 433.122 ± 2.61 μg TE/g (Figure 9). Similar activity was noticed in the sample GE3 429.74 ± 2.54 μg TE/g). Lowest antioxidant activity was observed in sample AE2 (397.63 ± 0.72 μg TE/g) (Figure 9). All the samples prepared with ethanol, as in the case of aqueous extracts, were significantly higher compared to control samples (*p* < 0.05). The ABTS radical scavenging capability using excipients and 50% ethanol (*v*/*v*) as a solvent of the test samples can be ranked, according to their average means, as follows: GE > BE > AE. 

A trend between test samples was observed comparing the results obtained with the DPPH and ABTS methods. Samples that were prepared in water had higher radical scavenging activity when they were extracted using *α*- or *β*-CD. The results gained using these two excipients were similar in both antioxidant methods. Samples prepared using ethanol and CDs by both ABTS and DPPH methods showed identical results. The highest antioxidant activity was found in samples prepared with *γ*-CD and the lowest in extracts that contained *α*-CD.

#### 3.2.3. Reducing Power Activity

The reducing capacity of a compound can be a significant indicator of its potential antioxidant activity. Compounds with reducing power indicate that they are electron donors and can reduce the oxidized intermediates of lipid peroxidation processes, so that they can act as primary and secondary antioxidants [52]. 

Total antioxidant activity, measured by the FRAP method, varied from 144.05 ± 0.73 (GE2) to 193.57 ± 1.94 (GE1) mg FE(II)/g dw in the test samples with excipients and 50% ethanol (*v*/*v*) as a solvent (Table 3). In aqueous samples extracted using different CDs as excipients, the highest antioxidant activity was observed in GW3 (143.37 ± 2.20 mg FE(II)/g dw) and the lowest—AW2 (106.45 ± 2.54 mg FE(II)/g dw). Examining the results obtained using FRAP assay, it was detected that only two samples—GE1 and AW2—had significant lower values compared with controls (Table 3).

In the literature, it was reported that red clover flowers have a low (0.0251 mg FE(II)/g dw) or even undetected reducing antioxidant power [53,54]. The test samples results obtained in this study were significantly higher than compared to controls. This means that it is very important to choose the right extraction conditions and solvent. However, the presence of phenolic compounds and their distribution in the red clover vary due to many factors—origin, cultivation influences, weather conditions, plant parts, growing stages, harvesting season, etc. [55]. Therefore, it would not be entirely appropriate to compare the results of different studies.

Some of the samples exhibited higher capacity in reducing ferric ion (Fe^3+^) to ferrous ion (Fe^2+^) than to scavenging free radicals. The use of *β*- or *γ*-CDs greatly increased the reducing power in both water and ethanol samples. Although the use of *γ*-CDs in ethanolic extracts gave a higher radical scavenging effect, aqueous extracts prepared with *γ*-CDs showed a higher reducing capacity. A similar trend was observed with *β*-CD. However, extracts prepared using *β*-CD and water showed stronger radical scavenging, but ethanolic samples extracted with *β*-CD had better reducing power. Even though aqueous extracts prepared using *α*-CD had a high radical scavenging activity, in both ethanol and water they had lower reducing properties compared to other test samples prepared using *β*- or *γ*-CDs. Once again, these results obtained with excipients could prove that using different CDs or/and solvent in the extraction media different compounds can be extracted, and the phenolic profile of the sample prepared with *α*-CD can be different from the samples prepared with *β*- or *γ*-CDs.

Comparing all antioxidant activity results obtained from three methods (FRAP, ABTS, DPPH), it was found that the CDs use increases antioxidant activity. This can be attributed not only to the higher yield of polyphenols and their improved diversity in the CD extracts, but also from the protection of the polyphenols from rapid oxidation by free radicals (during all treatment steps) due to CD encapsulation [56]. In this study, CD whose extracts were least affected by the solvent used (water or ethanol) was *β*-CD. 

#### 3.2.4. Post-Column ABTS Antioxidant Assay and RP-LC/PDA/MS Qualitative Analysis

The post-column detection of the reduction of the ABTS radical in relation to antioxidant content is reflected by the negative chromatogram at 650 nm. HPLC separation is coupled with rapid identification of antioxidative active compounds. The major advantages of post-column reaction methods are that the antioxidant activity of an individual compound can be measured and its contribution to the total activity of a complex mixture can be estimated [29]. 

The highest antioxidant activity using the post-column ABTS method was determined in the ethanolic GE1 sample—30.16 ± 1.21 mg TE/g and the lowest in the BE1 10.73 ± 0.43 mg TE/g ethanolic sample (Table 4). All the samples had statistically higher values compared with controls.

Results obtained from the post-column method show that using water the highest radical scavenging activity was in the BW1 sample (20.68 ± 0.83 mg TE/g) extracted using *β*-CD (Table 4). Much smaller results were obtained from the AW1 and GW1 samples. Ethanolic samples AE1 and GE1 had similar antioxidant properties, but BE1 antioxidant activity was almost three times lower. Therefore, samples obtained from aqueous *β*-CD assisted extraction had higher antioxidant activity than the same sample prepared in ethanol. Similar reverse correlation was observed in samples prepared with *α*- and *γ*-CDs.

**Figure 10:**
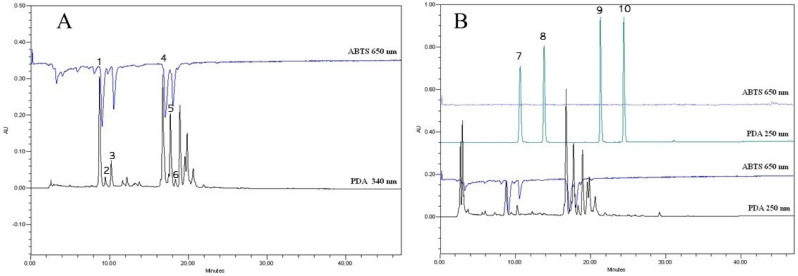
ABTS post-column chromatograms: (**A**) example profile of AW1 sample; (**B**) concentrated control sample profile with main isoflavones (7—daidzin; 8—genistin; 9—daidzein; 10—genistein) standards.

Figure 10, part A, shows an example chromatogram of sample AW1. Antioxidant activity signal strength is expressed as peaks of negative active compounds. It shows that the extract is rich in many different compounds, but not all of them have antioxidant activity. 

In chromatogram B (Figure 10), it is shown the results of concentrated control sample W1. It is widely reported in the literature [4,57,58] that red clover is rich in isoflavones, which have strong antioxidant properties. Therefore, it was decided to determine the antioxidant activity of isoflavones aglycones (daidzein (9) and genistein (10)) and glycosides (daidzin (7) and genistin (8)) in the extract using the ABTS post-column method.

In the study, we used known concentrations of isoflavones standards, and it was determined that isoflavones tested in the post-column method did not show antioxidant activity. The same isoflavones standard samples were investigated using the ABTS spectrophotometric method and the result was 134.64 ± 5.87 μg TE/g. This means that isoflavones need longer incubation time in the ABTS assay to reach equilibrium. It was reported in the literature that whenusing an ABTS assay not all the compounds react quickly, so longer incubation is needed [59,60]. Using spectrophotometric method incubation time was 30 min, but in column it was much shorter, which resulted in the absence of antioxidant activity. 

Using HPLC-DAD, only two compounds were identified in the red clover profile—caffeic acid (2) and hyperoside (4). To determine all the compounds that gave the highest antioxidant activity to the red clover extract (determined by the post-column method), it was decided to use LC-MS. It was determined that peak **1** (*m*/*z* 295.0 [M-H]^–^) was caffeoylmalic acid, peak **3** (*m*/*z* 358.0 [M-H]^–^) cis-clovamide, peak **5** (*m*/*z* 505.1 [M-H]^–^) quercetin-O-hexoside-acetate, and peak **6** (*m*/*z* 447.0 [M-H]^–^) kaempferol-O-hexoside, by comparing with the data found in the literature and databases [61].

According to ABTS post-column chromatogram (Figure 10A), caffeoylmalic acid gives the highest antioxidant respond compared to other compounds in the extract. Moreover, a quite high response was detected from hyperoside as well. 

### 3.3. Correlation with Antioxidant Activities and Phytochemical Contents

In this study, four methods used to quantify the antioxidant activity of the samples prepared with CDs-assisted extraction. Furthermore, all the methods presented a significant correlation between them, with high values in the Spearman correlation coefficient. Numerous studies have also reported this correlation [43,55].

In the aqueous samples extracted, α-CD significant positive correlations were found between total phenolic content and DPPH and FRAP assays. Similar positive correlations were observed between total flavonoid content and DPPH and FRAP assays. ABTS assay correlations with total phenolic and flavonoid content were moderate (Table 5).

In ethanolic extracts, total phenolic and flavonoid content had significant positive correlations with DPPH and ABTS assays; in these samples, FRAP assay correlation was very weak (Table 5). The antioxidant ability of polyphenols, including mainly flavonoids, seemed to be an important factor dictating the free radical scavenging capacity of red clover flower ethanolic extracts prepared with α-CD.

The most statistically significant correlations between the antioxidant assay and phenolic and flavonoids content were observed in aqueous test samples prepared with *β*-CD (Table 6). Even though flavonoid content presented nonsignificant moderate correlation with the DPPH assay, it demonstrated strong correlations with ABTS and FRAP assays.

In ethanolic extracts, correlations between flavonoid content and antioxidant assays were from weak (DPPH) to moderate (ABTS and FRAP). However, in ethanolic samples, the total phenolic content statistically significant correlated with DPPH and FRAP assays (Table 6).

Chelating power in aqueous samples prepared with *γ*-CD had a significant correlation between flavonoids, but nonsignificant with total phenolic content. Total phenolic content pointed to a significant correlation with DPPH (Table 7).

In ethanolic extracts, the only statistically significant correlation was between total phenolic content and the DPPH assay.

All the samples prepared with excipients demonstrated a strong relationship between total phenolic content and DPPH assay. FRAP assay in all the aqueous samples (except samples prepared using *γ*-CD) had a significant correlation with total phenolic and total flavonoid contents.

## 4. Conclusions

The observed levels of phenolics, total flavonoid content and the antioxidant properties of red clover flowers extract indicate that it can be used as a natural source of biologically active components in the human nutrition, as well as in pharmaceutical industry.

Using *α*-, *β*-, and *γ*-CDs during aqueous or ethanolic red clover extraction enhanced antioxidant activity in samples. Either CD extracted different compounds in extraction media that had different activities. The total phenolic contents in samples of the extracts varied from 54.12 ± 0.46 (GE3) to 34.38 ± 0.63 (AE2) mg GA/g dw when the solvent was 50% (*v*/*v*) ethanol and 44.78 ± 0.92 (BW1) to 32.31 ± 0.63 (BW3) mg GA/g dw when the solvent was water. All the samples that were prepared with excipients, despite the solvent used, had illustrated higher total content of phenolic compounds compared to control samples prepared without CDs. The total flavonoid content results were entirely synchronous with those of the total phenolic. It was successfully shown that samples with a high level of phenolic content also contain flavonoids in great amount.

Samples that were prepared in water had a higher radical scavenging activity than when they were extracted using *α*- or *β*-CD. The results gained using these two excipients were similar in both antioxidant methods. Samples prepared using ethanol and CDs by both ABTS and DPPH methods showed identical results. The highest antioxidant activity was found in samples prepared with *γ*-CD and the lowest in extracts that contained *α*-CD. Some of the samples exhibited higher capacity in reducing ferric ion to ferrous ion than to scavenging free radicals: samples with *β*- or *γ*-CDs greatly increased reducing power in both water and ethanol samples. Although the use of *γ*-CDs in ethanolic extracts gave a higher radical scavenging effect, aqueous extracts prepared with *γ*-CDs showed a higher reducing capacity. Using the post-column method, it is important to determine if active compounds react slowly with the ABTS assay, or quickly to optimize the method and obtain quality results. The most statistically significant correlations between the antioxidant assay and phenolic and flavonoids content were observed in aqueous test samples prepared with *β*-CD. Additionally, samples prepared using the *β*-CD assisted method in water showed the highest phenolic and flavonoid content as well as antioxidant activity. However, using *γ*-CD increased antioxidant activity and phenolic compounds in 50% ethanol samples. Thus, different CDs increase the amount of extracted phenolic compounds, which have strong antioxidant properties, in the desired safe solvent, making CDs a great and safe for food tool for obtaining rich extracts that can be used in the pharmaceutical industry for nutraceuticals production.

## Figures and Tables

**Table 1:** Samples list and preparation conditions.

Sample Name *	Method	Solvent	Excipients
AW1	Reflux (1)	water	*α*-CD
AW2	Ultrasound (10 min) combined with reflux (2)	water	*α*-CD
AW3	Ultrasound (30 min) combined with reflux (3)	water	α-CD
AE1	Reflux (1)	ethanol	α-CD
AE2	Ultrasound (10 min) combined with reflux (2)	ethanol	α-CD
AE3	Ultrasound (30 min) combined with reflux (3)	ethanol	α-CD
BW1	Reflux (1)	water	*β*-CD
BW2	Ultrasound (10 min) combined with reflux (2)	water	*β*-CD
BW3	Ultrasound (30 min) combined with reflux (3)	water	*β*-CD
BE1	Reflux (1)	ethanol	*β*-CD
BE2	Ultrasound (10 min) combined with reflux (2)	ethanol	*β*-CD
BE3	Ultrasound (30 min) combined with reflux (3)	ethanol	*β*-CD
GW1	Reflux (1)	water	*γ*-CD
GW2	Ultrasound (10 min) combined with reflux (2)	water	*γ*-CD
GW3	Ultrasound (30 min) combined with reflux (3)	water	*γ*-CD
GE1	Reflux (1)	ethanol	*γ*-CD
GE2	Ultrasound (10 min) combined with reflux (2)	ethanol	*γ*-CD
GE3	Ultrasound (30 min) combined with reflux (3)	ethanol	*γ*-CD
W1	Reflux (1)	water	-
W2	Ultrasound (10 min) combined with reflux (2)	water	-
W3	Ultrasound (30 min) combined with reflux (3)	water	-
E1	Reflux (1)	ethanol	-
E2	Ultrasound (10 min) combined with reflux (2)	ethanol	-
E3	Ultrasound (30 min) combined with reflux (3)	ethanol	-

* 1-3 means different conditions for sample preparation: (1) reflux; (2) ultrasound (10 min) combined with reflux; (3) ultrasound (30 min) combined with reflux (3).

**Table 2:** Flavonoid content (mg RU/g dw) of *Trifolium pratense* L. flowers.

Samples Codes *	Total Flavonoid Content (mg RU/g dw) **
W1	18.17 ± 0.67
W2	18.51 ± 0.67
W3	19.00 ± 0.39
AW1	20.35 ± 0.17 ^a,b,c^
AW2	20.57 ± 0.18 ^a,b,c^
AW3	20.57 ± 0.24 ^a,b,c^
BW1	21.07 ± 0.20 ^a,b,c^
BW2	21.74 ± 0.12 ^a,b,c^
BW3	21.47 ± 0.23 ^a,b,c^
GW1	19.56 ± 0.10 ^a^
GW2	18.42 ± 0.38 ^a^
GW3	17.95 ± 0.28
E1	20.51 ± 0.39
E2	21.16 ± 0.18
E3	19.81 ± 0.27
AE1	21.13 ± 0.13 ^d,f^
AE2	21.18 ± 0.24 ^d,f^
AE3	20.98 ± 0.28 ^d,f^
BE1	21.11 ± 0.28 ^d,f^
BE2	20.82 ± 0.16 ^f^
BE3	20.84 ± 0.38 ^f^
GE1	20.93 ± 0.23 ^d,f^
GE2	20.91 ± 0.31 ^d,f^
GE3	21.09 ± 0.19 ^d,f^

* Samples codes are provided in Table 1. ** Results are mean values (n = 3); the ± values relative standard deviations SD. ^a^ *p* < 0.05 vs. W1; ^b^ *p* < 0.05 vs. W2; ^c^ *p* < 0.05 vs. W3; ^d^ *p* < 0.05 vs. E1; ^f^ *p* < 0.05 vs. E3. The control in water is compared only with samples prepared in water. Moreover, controls prepared in ethanol are compared only with ethanol samples.

**Table 3:** Ferric Reducing Antioxidant Power (FRAP) values in samples prepared with excipients.

Samples Codes *	FRAP, mg FE(II)/g dw **
E1	152.93 ± 1.93
E1	144.09 ± 1.47
E3	141.05 ± 1.94
AE1	167.78 ± 3.19 ^d,e,f^
AE2	161.60 ± 1.94 ^d,e,f^
AE3	161.35 ± 1.94 ^d,e,f^
BE1	188.84 ± 5.13 ^d,e,f^
BE2	162.07 ± 2.93 ^d,e,f^
BE3	191.92 ± 0.73 ^d,e,f^
GE1	193.57 ± 1.94 ^d,e,f^
GE2	144.05 ± 0.73 ^f^
GE3	179.62 ± 1.94 ^d,e,f^
W1	109.92 ± 2.71
W2	106.58 ± 1.12
W3	102.60 ± 1.59
AW1	120.79 ± 1.27 ^a,b,c^
AW2	106.45 ± 2.54 ^c^
AW3	114.36 ± 0.73 ^a,b,c^
BW1	142.10 ± 2.54 ^a,b,c^
BW2	121.38 ± 1.47 ^a,b,c^
BW3	116.43 ± 2.93 ^a,b,c^
GW1	112.71 ± 0.73 ^a,b,c^
GW2	136.44 ± 0.41 ^a,b,c^
GW3	143.37 ± 2.20 ^a,b,c^

* Samples codes are provided in Table 1. ** Results are mean values (n = 3); the ± values relative standard deviations SD. ^a^ *p* < 0.05 vs. W1; ^b^ *p* < 0.05 vs. W2; ^c^ *p* < 0.05 vs. W3; ^d^ *p* < 0.05 vs. E1; ^e^ *p* < 0.05 vs. E2; ^f^ *p* < 0.05 vs. E3. The control in water is compared only with samples prepared in water. Moreover, controls prepared in ethanol are compared only with ethanol samples.

**Table 4:** ABTS radical scavenging activity of red clover blossom extracts with 50% (*v*/*v*) of ethanol or water as a solvent with excipients.

Samples Codes *	ABTS Radical Scavenging, mg TE/g dw **
AW1	11.93 ± 0.48 ^b^
AE1	32.68 ± 1.31 ^a^
BW1	20.68 ± 0.83 ^b^
BE1	10.73 ± 0.43 ^a^
GW1	12.67 ± 0.51 ^b^
GE1	30.16 ± 1.21 ^a^
W1	8.35 ± 0.33
E1	9.38 ± 0.38

* Samples codes are provided in Table 1. ** ^a^ *p* < 0.05 vs. E1; ^b^ *p* < 0.05 vs. W1. The control in water is compared only with samples prepared in water. Moreover, controls prepared in ethanol are compared only with ethanol samples.

**Table 5:** Correlation between antioxidant activity and total phenolic and total flavonoid content of the red clover aerial extracts prepared with *α*-CD.

Assays	Correlations of Aqueous Extracts		Correlations of Ethanolic Extracts	
	Total Phenolic	Total Flavonoid	Total Phenolic	Total Flavonoid
DPPH radical scavenging activity	0.989 **	0.979 **	0.897 **	0.999 **
ABTS radical scavenging activity	0.447	0.500	0.986 **	0.830 **
Ferric Reducing Antioxidant Power	0.800 **	0.835 **	0.079	0.338

** Correlation is significant at the 0.01 level (2-tailed).

**Table 6:** Correlation between antioxidant activity and total phenolic and total flavonoid content of the red clover aerial extracts prepared with *β*-CD.

Assays	Correlations of Aqueous Extracts		Correlations of Ethanolic Extracts	
	Total Phenolic	Total Flavonoid	Total Phenolic	Total Flavonoid
DPPH radical scavenging activity	0.999 **	0.558	0.979 **	0.344
ABTS radical scavenging activity	0.938 **	0.832 **	0.424	0.545
Ferric Reducing Antioxidant Power	0.939 **	0.830 **	0.998 **	0.480

** Correlation is significant at the 0.01 level (2-tailed).

**Table 7:** Correlation between antioxidant activity and total phenolic and total flavonoid content of the red clover aerial extracts prepared with γ-CD.

Assays	Correlations of Aqueous Extracts		Correlations of Ethanolic Extracts	
	Total Phenolic	Total Flavonoid	Total Phenolic	Total Flavonoid
DPPH radical scavenging activity	0.999 **	0.293	0.836 **	0.570
ABTS radical scavenging activity	0.307	0.936 **	0.083	0.450
Ferric Reducing Antioxidant Power	0.316	0.998 **	0.024	0.353

** Correlation is significant at the 0.01 level (2-tailed).

## Data Availability

Not applicable.

## References

[1] Dixon R.A. (2004). Phytoestrogens. Annu. Rev. Plant Biol..

[2] Khorasani Esmaeili A., Mat Taha R., Mohajer S., Banisalam B. (2015). Antioxidant Activity and Total Phenolic and Flavonoid Content of Various Solvent Extracts from In Vivo and In Vitro Grown Trifolium Pratense L. (Red Clover). Biomed Res. Int..

[3] Booth N.L., Overk C.R., Yao P., Burdette J.E., Nikolic D., Chen S., Bolton J.L., van Breemen R.B., Pauli G.F., Farnsworth N.R. (2006). The Chemical and Biologic Profile of a Red Clover ( Trifolium Pratense L.) Phase II Clinical Extract. J. Altern. Complement. Med..

[4] Tava A., Pecio Ł., Lo Scalzo R., Stochmal A., Pecetti L. (2019). Phenolic Content and Antioxidant Activity in Trifolium Germplasm from Different Environments. Molecules.

[5] Rietjens I.M.C.M., Louisse J., Beekmann K. (2017). The Potential Health Effects of Dietary Phytoestrogens. Br. J. Pharmacol..

[6] Atkinson C., Compston J.E., Day N.E., Dowsett M., Bingham S.A. (2004). The Effects of Phytoestrogen Isoflavones on Bone Density in Women: A Double-Blind, Randomized, Placebo-Controlled Trial. Am. J. Clin. Nutr..

[7] Abdi F., Alimoradi Z., Haqi P., Mahdizad F. (2016). Effects of Phytoestrogens on Bone Mineral Density during the Menopause Transition: A Systematic Review of Randomized, Controlled Trials. Climacteric.

[8] Marini H., Minutoli L., Polito F., Bitto A., Altavilla D., Atteritano M., Gaudio A., Mazzaferro S., Frisina A., Frisina N. (2007). Effects of the Phytoestrogen Genistein on Bone Metabolism in Osteopenic Postmenopausal Women: A Randomized Trial. Ann. Intern. Med..

[9] Tucak M., Čupić T., Horvat D., Popović S., Krizmanić G., Ravlić M. (2020). Variation of Phytoestrogen Content and Major Agronomic Traits in Alfalfa (Medicago Sativa l.) Populations. Agronomy.

[10] Tapas A., Sakarkar D., Kakde R. (2008). Flavonoids as Nutraceuticals: A Review. Trop. J. Pharm. Res..

[11] Khan A.K., Kousar S., Tungmunnithum D., Hano C., Abbasi B.H., Anjum S. (2021). Nano-Elicitation as an Effective and Emerging Strategy for in Vitro Production of Industrially Important Flavonoids. Appl. Sci..

[12] Dobrinas S., Soceanu A., Popescu V., Carazeanu Popovici I., Jitariu D. (2021). Relationship between Total Phenolic Content, Antioxidant Capacity, Fe and Cu Content from Tea Plant Samples at Different Brewing Times. Processes.

[13] Piluzza G., Bullitta S. (2011). Correlations between Phenolic Content and Antioxidant Properties in Twenty-Four Plant Species of Traditional Ethnoveterinary Use in the Mediterranean Area. Pharm. Biol..

[14] Wojdyło A., Oszmiański J., Czemerys R. (2007). Antioxidant Activity and Phenolic Compounds in 32 Selected Herbs. Food Chem..

[15] Cheynier V. (2012). Phenolic Compounds: From Plants to Foods. Phytochem. Rev..

[16] Luna-Guevara M.L., Luna-Guevara J.J., Hernández-Carranza P., Ruíz-Espinosa H., Ochoa-Velasco C.E. (2018). Phenolic Compounds: A Good Choice Against Chronic Degenerative Diseases. Stud. Nat. Prod. Chem..

[17] Nakamoto M., Otsuka R., Nishita Y., Tange C., Tomida M., Kato Y., Imai T., Sakai T., Ando F., Shimokata H. (2018). Soy Food and Isoflavone Intake Reduces the Risk of Cognitive Impairment in Elderly Japanese Women. Eur. J. Clin. Nutr..

[18] Miller K.A., Frankel F., Takahashr H., Vance N., Stiegerwald C., Edelstein S. (2016). Collected Literature on Isoflavones and Chronic Diseases. Cogent Food Agric..

[19] Hanganu D., Benedec D., Vlase L., Olah N., Damian G., Silaghi-Dumitrescu R., Moț A.C., Toma C.C. (2017). Polyphenolic Profile and Antioxidant and Antibacterial Activities from Two Trifolium Species. Farmacia.

[20] Petrovic M.P., Stankovic M.S., Andelkovic B.S., Babic S.Z., Zornic V.G., Vasiljevic S.L., Dajic-Stevanovic Z.P. (2016). Quality Parameters and Antioxidant Activity of Three Clover Species in Relation to the Livestock Diet. Not. Bot. Horti Agrobot. Cluj-Napoca.

[21] Cheynier V., Tomas-Barberan F.A., Yoshida K. (2015). Polyphenols: From Plants to a Variety of Food and Nonfood Uses. J. Agric. Food Chem..

[22] Kazlauskaite J.A., Ivanauskas L., Bernatoniene J. (2021). Cyclodextrin-Assisted Extraction Method as a Green Alternative to Increase the Isoflavone Yield from Trifolium Pratensis l. Extract. Pharmaceutics.

[23] Khoddami A., Wilkes M.A., Roberts T.H. (2013). Techniques for Analysis of Plant Phenolic Compounds. Molecules.

[24] Kaurinovic B., Popovic M., Vlaisavljevic S., Schwartsova H., Vojinovic-Miloradov M. (2012). Antioxidant Profile of *Trifolium Pratense* L. Molecules.

[25] Tsamo D.L.F., Tamokou J.-D.-D., Kengne I.C., Ngnokam C.D.J., Djamalladine M.D., Voutquenne-Nazabadioko L., Ngnokam D. (2021). Antimicrobial and Antioxidant Secondary Metabolites from Trifolium Baccarinii Chiov. (Fabaceae) and Their Mechanisms of Antibacterial Action. Biomed Res. Int..

[26] Vlaisavljević S., Kaurinović B., Popović M., Vasiljević S. (2017). Profile of Phenolic Compounds in Trifolium Pratense L. Extracts at Different Growth Stages and Their Biological Activities. Int. J. Food Prop..

[27] Chaves N., Santiago A., Alías J.C. (2020). Quantification of the Antioxidant Activity of Plant Extracts: Analysis of Sensitivity and Hierarchization Based on the Method Used. Antioxidants.

[28] Raudonis R., Raudone L., Jakstas V., Janulis V. (2012). Comparative Evaluation of Post-Column Free Radical Scavenging and Ferric Reducing Antioxidant Power Assays for Screening of Antioxidants in Strawberries. J. Chromatogr. A.

[29] Marksa M., Radušienė J., Jakštas V., Ivanauskas L., Marksienė R. (2016). Development of an HPLC Post-Column Antioxidant Assay for Solidago Canadensis Radical Scavengers. Nat. Prod. Res..

[30] Chaudhari S.P., Patil P.S. (2012). Pharmaceutical Excipients: A Review. Int. J. Adv. Pharmacy, Biol. Chem..

[31] Otero-Espinar F.J., Torres-Labandeira J.J., Alvarez-Lorenzo C., Blanco-Méndez J. (2010). Cyclodextrins in Drug Delivery Systems. J. Drug Deliv. Sci. Technol..

[32] Zhao M., Wang H., Yang B., Tao H. (2010). Identification of Cyclodextrin Inclusion Complex of Chlorogenic Acid and Its Antimicrobial Activity. Food Chem..

[33] Challa R., Ahuja A., Ali J., Khar R.K. (2005). Cyclodextrins in Drug Delivery: An Updated Review. AAPS PharmSciTech.

[34] Loftsson T., Brewster M.E. (1996). Pharmaceutical Applications of Cyclodextrins. 1. Drug Solubilization and Stabilization. J. Pharm. Sci..

[35] Conceição J., Adeoye O., Cabral-Marques H.M., Lobo J.M.S. (2018). Cyclodextrins as Excipients in Tablet Formulations. Drug Discov. Today.

[36] Saviranta N.M.M., Julkunen-Tiitto R., Oksanen E., Karjalainen R.O. (2010). Leaf Phenolic Compounds in Red Clover (Trifolium Pratense L.) Induced by Exposure to Moderately Elevated Ozone. Environ. Pollut..

[37] Urbonavičiute A., Jakštas V., Kornyšova O., Janulis V., Maruška A. (2006). Capillary Electrophoretic Analysis of Flavonoids in Single-Styled Hawthorn (Crataegus Monogyna Jacq.) Ethanolic Extracts. J. Chromatogr. A.

[38] Re R., Pellegrini N., Proteggente A., Pannala A., Yang M., Rice-Evans C. (1999). Antioxidant Activity Applying an Improved ABTS Radical Cation Decolorization Assay. Free Radic. Biol. Med..

[39] Agourram A., Ghirardello D., Rantsiou K., Zeppa G., Belviso S., Romane A., Oufdou K., Giordano M. (2013). Phenolic Content, Antioxidant Potential, and Antimicrobial Activities of Fruit and Vegetable by-Product Extracts. Int. J. Food Prop..

[40] Tsao R., Papadopoulos Y., Yang R., Chris Young J., Mcrae K. (2006). Isoflavone Profiles of Red Clovers and Their Distribution in Different Parts Harvested at Different Growing Stages. J. Agric. Food Chem..

[41] Butkute B., Lemežiene N., Dabkevičiene G., Jakštas V., Vilčinskas E., Janulis V. (2014). Source of Variation of Isoflavone Concentrations in Perennial Clover Species. Pharmacogn. Mag..

[42] Booth N.L., Overk C.R., Yao P., Totura S., Deng Y., Hedayat A.S., Bolton J.L., Pauli G.F., Farnsworth N.R. (2006). Seasonal Variation of Red Clover (Trifolium Pratense L., Fabaceae) Isoflavones and Estrogenic Activity. J. Agric. Food Chem..

[43] Kicel A., Wolbiś M. (2013). Phenolic Content and Dpph Radical Scavenging Activity of the Flowers and Leaves of Trifolium Repens. Nat. Prod. Commun..

[44] Chavenetidou M.A., Pankou C.I., Tziouvalekas M.S. (2021). A Qualitative and Quantitative Analysis of Extractives from the Species Trifolium Pratense l. In Three Different Solvents. Agric. For..

[45] Gikas E., Alesta A., Economou G., Karamanos A., Tsarbopoulos A. (2008). Determination of Isoflavones in the Aerial Part of Red Clover by HPLC-Diode Array Detection. J. Liq. Chromatogr. Relat. Technol..

[46] Singh P., Arif Y., Bajguz A., Hayat S. (2021). The Role of Quercetin in Plants. Plant Physiol. Biochem..

[47] Brewer M.S. (2011). Natural Antioxidants: Sources, Compounds, Mechanisms of Action, and Potential Applications. Compr. Rev. Food Sci. Food Saf..

[48] Yahyaoui M., Ghazouani N., Sifaoui I., Abderrabba M. (2017). Comparison of the Effect of Various Extraction Methods on the Phytochemical Composition and Antioxidant Activity of Thymelaea Hirsuta L. Aerial Parts in Tunisia. Biosci. Biotechnol. Res. Asia.

[49] Shiozawa R., Inoue Y., Murata I., Kanamoto I. (2018). Effect of Antioxidant Activity of Caffeic Acid with Cyclodextrins Using Ground Mixture Method. Asian J. Pharm. Sci..

[50] Chao J., Wang H., Zhao W., Zhang M., Zhang L. (2012). Investigation of the Inclusion Behavior of Chlorogenic Acid with Hydroxypropyl-β-Cyclodextrin. Int. J. Biol. Macromol..

[51] Akbaribazm M., Khazaei M.R., Khazaei M. (2020). Phytochemicals and Antioxidant Activity of Alcoholic/Hydroalcoholic Extract of Trifolium Pratense. Chinese Herb. Med..

[52] Benslama A., Harrar A. (2016). Free Radicals Scavenging Activity and Reducing Power of Two Algerian Sahara Medicinal Plants Extracts. Int. J. Herb. Med..

[53] Jakubczyk K., Łukomska A., Gutowska I., Kochman J., Janił J., Janda K. (2021). Edible Flowers Extracts as a Source of Bioactive Compounds with Antioxidant Properties—in Vitro Studies. Appl. Sci..

[54] Tundis R., Marrelli M., Conforti F., Tenuta M.C., Bonesi M., Menichini F., Loizzo M.R. (2015). Trifolium Pratense and t. Repens (Leguminosae): Edible Flower Extracts as Functional Ingredients. Foods.

[55] Horvat D., Tucak M., Viljevac Vuletić M., Čupić T., Krizmanić G., Kovačević Babić M. (2020). PHENOLIC CONTENT AND ANTIOXIDANT ACTIVITY OF THE CROATIAN RED CLOVER GERMPLASM COLLECTION. Poljoprivreda.

[56] Cai R., Yuan Y., Cui L., Wang Z., Yue T. (2018). Cyclodextrin-Assisted Extraction of Phenolic Compounds: Current Research and Future Prospects. Trends Food Sci. Technol..

[57] Křížová L., Dadáková K., Kašparovská J., Kašparovský T. (2019). Isoflavones. Molecules.

[58] Szeja W., Grynkiewicz G., Rusin A. (2016). Isoflavones, Their Glycosides and Glycoconjugates. Synthesis and Biological Activity. Curr. Org. Chem..

[59] Zheng L., Zhao M., Xiao C., Zhao Q., Su G. (2016). Practical Problems When Using ABTS Assay to Assess the Radical-Scavenging Activity of Peptides: Importance of Controlling Reaction PH and Time. Food Chem..

[60] Zielinska D., Szawara-Nowak D., Zielinski H. (2007). Comparison of Spectrophotometric and Electrochemical Methods for the Evaluation of the Antioxidant Capacity of Buckwheat Products after Hydrothermal Treatment. J. Agric. Food Chem..

[61] Magdalena Maciejewska-Turska G.Z. (2022). In-Depth Phytochemical and Biological Studies on Potential AChE Inhibitors in Red and Zigzag Clover Dry Extracts Using Reversed – Phase Liquid Chromatography ( RP-LC ) Coupled with Photodiode Array ( PDA ) and Electron Spray Ionization-Quadrupole / Time O. Food Chem..

